# Proceedings of the Illinois State Medical Society for 1865

**Published:** 1865-05

**Authors:** 


### Proceedings of Societies.

### PROCEEDINGS OF THE ILLINOIS STATE MEDICAL SOCIETY FOR 1865.

The regular Annual Meeting of the Illinois State Medical Society assembled in Royce's Hall, Bloomington, May 2, 1865, at 10½ o'clock A.M. The President being absent, Dr. J. M. Steele, of Grandview, Vice-President, took the Chair.

Dr. T. F. Worrell, Chairman of the Committee of Arrangements, welcomed the members of the Society to the City of Bloomington, and the hospitalities of its citizens, in an eloquent manner.

The Committee also reported as present the following delegates and permanent members, viz.:—

Drs. N. S. Davis, J. H. Hollister, J. S. Jewell, and E. L. Holmes, of Chicago, Cook Co.

Drs. W. J. Ballard, H. C. Luce, Z. L. Hoover, C. R. Parks, T. F. Worrell, D. L. Crist, E. R. Roe, T. P. Rogers, of Bloomington, McLean Co.

Dr. T. D. Fisher, of Leroy, McLean Co.

Dr. H. Noble, of Heyworth, McLean Co.

Drs. J. H. Tyler, D. W. Edmiston, W. W. Adams, C. Goodbrake, of Clinton, DeWitt Co.

Dr. L. T. Hewins, of Loda, Iroquois Co.

Dr. A. L. McArthur, of Joliet, Will Co.

Dr. R. E. McVey, of Waverly, Morgan Co.

Drs. S. T. Trowbridge, S. McBride, and E. W. Moore, of Decatur, Macon Co.

Dr. F. B. Haller, of Vandalia, Fayette Co.

Dr. N. Wright, of Chatham, Sangamon Co.

Dr. J. M. Steele, of Grandview, Edgar Co.

Dr. J. W. Redden, of Shawneetown, Gallatin Co.

The election of new members being in order, the following were nominated and unanimously elected:—

Dr. D. T. Whitnell, of Vienna, Johnson Co.
" A. N. Lodge, of Marion, Vermilion Co.
" J. J. Samuels, do do.
" W. H. Veatch, of Pawnee, Sangamon Co.
" H. P. Merriman, of Chicago, Cook Co.
" J. E. Owens, do do.
" S. M. Mitchell, of Attilla, Williamson Co.
" R. T. Higgins, of Vandalia, Fayette Co.
" W. A. Elder, of Bloomington, McLean Co.
" D. A. Crist, do do.

The following letter was received from the President, Dr. A. H. Luce, and read by the Secretary:—

Bloomington, *May 2d*, 1865.

Dr. C. R. Park, *Secretary of Ill. State Med. Soc'y*,

Dear Sir:—Owing to a severely painful affliction, I shall be compelled to forego the pleasure of attending the present meeting of the Society, and discharging the last duty of my honorable office. Hoping that you will have a pleasant and profitable Session; with my best wishes for the prosperity of our Association, and for the good of its Members, individually,

I remain, yours truly, A. H. Luce,

*President Ill. State Med. Soc'y.*

The following Committee of one from each county represented, was appointed to nominate officers, and fill the Standing Committees, for the ensuing year, viz.:—

Dr. N. S. Davis, of Cook Co.
" J. M. Steele, of Edgar Co.
" A. L. McArthur, of Will Co.
" D. W. Edmiston, of DeWitt Co.
" S. McBride, of Macon Co.
" L. T. Hewins, of Iroquois Co.
" W. J. Ballard, of McLean Co.
" F. B. Haller, of Fayette Co.
" N. Wright, of Sangamon Co.
" R. E. McVey, of Morgan Co.
" D. T. Whitnell, of Johnston Co.
" J. W. Redden, of Gallatin Co.

After a few minutes recess, Dr. R. E. McVey, Chairman of the Nominating Committee, reported the following officers for the ensuing year, viz.:—

*For President*—Dr. J. M. Steele, of Grandview.
*Vice-President*—Dr. F. B. Haller, of Vandalia.
2*d Vice-President*—Dr. C. Goodbrake, of Clinton.
*Treasurer*—J. H. Hollister, of Chicago.
*Permanent-Secretary*—N. S. Davis, of Chicago.
*Assistant-Secretary*—W. J. Chenowith, of Decatur.

On motion, the report was accepted and unanimously adopted.

Drs. A. L. McArthur and S. T. Trowbridge were appointed a committee to conduct the newly elected officers to their places. Dr. McArthur introduced the President elect in a very appropriate manner. Dr. Steele, in taking the Chair, thanked the Society for the honor conferred upon him.

On motion, Dr. Young, of Ohio, was invited to take a seat in the Society, as a member by invitation.

On motion of Dr. J. H. Hollister, all members of the profession from other States, who might be present, were invited to participate in the meeting, as members by invitation.

Dr. J. H. Hollister offered the following preamble and resolution:—

*Whereas*, The members of the Illinois State Medical Society have learned, with deep regret, that their esteemed President, Dr. A. H. Luce, is unable to be present, to preside over the deliberations of this Annual Meeting of the Society, and deeply sympathize with him in his affliction; therefore,

*Resolved*, That a committee of five be appointed to express to him, in reply to his communication, the assurances of our unwavering professional regard, and personal esteem.

They were adopted, and the following gentlemen appointed to constitute the committee:—Drs. J. H. Hollister, S. T. Trowbridge, C. Goodbrake, L. T. Hewins, and N. S. Davis.

An invitation to visit the State Normal University, was received from the Principal of that institution, which was accepted.

The reports of Standing Committees being called for in order, no communications of any kind were received from any of them.

On motion, adjourned, until 2 o'clock P.M.

AFTERNOON SESSION.

The Society met at 2 o'clock P.M., the President, Dr. J. M. Steele, in the chair.

The report on "Spotted Fever," being the special order of business, Dr. J. S. Jewell gave an abstract of his report on that subject, which gave rise to an interesting discussion, participated in by Drs. A. L. McArthur, J. H. Hollister, S. T. Trowbridge, J. S. Jewell, C. Goodbrake, D. T. Whitnell, L. T. Hewins, T. P. Rogers, and N. S. Davis. It was evident, from the facts elicited during this discussion, either that several distinct diseases had been included under the name of Spotted Fever, or Cerebro-Spinal Meningitis, or that the disease is capable of arising from different causes, and under widely varying circumstances. In one locality in Michigan, its prevalence was alleged to have been directly connected with an immense quantity of decaying potatoes. In another locality, in this State, its origin was attributed to an extensive accumulation of the refuse cane, or sorghum, scattered over several acres of ground, and undergoing decomposition. By those who had served as medical officers in the army, it had been observed to occur most among those encamped in highly malarious localities. It was particularly frequent among the soldiers encamped on the Yazoo River. On the other hand, cases are reported to have occurred in localities supposed to be wholly destitute of malaria.

On motion, Dr. Jewell was requested to complete his report, and transmit it to the Committee of Publication.

Dr. R. E. McVey, of Waverly, read a paper, giving an account of an epidemic erysipelatous fever, which had occurred in his neighborhood. The subject of this paper elicited considerable discussion, after which it was referred to the Committee of Publication.

Dr. Hollister, in behalf of the committee to whom was referred the letter of the late President of the Society, Dr. A. H. Luce, reported the following:—

Bloomington, Ill., *May 2d*, 1865.

Dr. A. H. Luce, *President of the Ill. State Med. Soc'y:*

Dear Sir,—The undersigned were appointed a special committee of the State Medical Society, to reply to your communication, received this morning, and express to you our sincere regret that your bodily infirmity prevents you from presiding over the opening exercises of this Annual Meeting. We are instructed by the Society to express to you its sentiments of regard, in the following resolutions:—

*Resolved*, That the members of the Illinois State Medical Society have ever regarded the name and professional standing of Dr. A. H. Luce as a credit to our profession; that we have ever cherished for him sentiments of profound respect, both for his professional attainments and moral worth, and that our esteem was manifested in proffering to him the honor of the presidency of our State Society, as the most appropriate method by which our confidence could be expressed.

*Resolved*, That those sentiments of regard are *to-day unchanged;* that he has our sincere sympathy in his affliction, and, that we counsel and urge that, with the consciousness of his *worth* and *innocence*, he bear up bravely in his trials, assured that in the medical profession he has *true* and *trusty friends*, as *numerous* as are associates in it.

*Resolved*, That Drs. H. Noble, C. Goodbrake, and T. F. Worrell, be a committee, to convey to him this answer to his communication.

The above resolutions, unanimously expressing the sense of the Society, are placed by us in the hands of the committee appointed to convey them to you. And we desire to remain, with unshaken confidence and esteem, your brothers in the medical profession,

JOHN H. HOLLISTER,
S. T. TROWBRIDGE,
C. GOODBRAKE, } Committee.
L. T. HEWINS,
N. S. DAVIS,

On motion of Dr. A. L. McArthur, the report of the committee was accepted and adopted.

Dr. N. S. Davis, Permanent-Secretary, presented the following report, which was accepted, and referred to the Committee of Publication:—

REPORT OF THE PERMANENT-SECRETARY AND COMMITTEE OF PUBLICATION.

Soon after the close of the last Annual Meeting of the Society, the undersigned notified the chairmen of all the committees of their appointment, and of the subjects assigned to them for investigation. About four months since, he also caused to be published in the two Medical Journals in this State, a notice of the time and place of the present Annual Meeting, specifying, also, the committees expected to report at the same.

Being furnished by the Treasurer with a list of the names of all such members as had paid their annual assessment for 1864, copies of the printed Transactions, for that year, were mailed to each, with prepayment of postage.

In behalf of the Publishing Committee, he would state that, as soon as the several reports and papers could be collected together, the publication was commenced in the same manner as during the two preceding years, namely, in such a way as to reduce the cost to the paper and presswork. Still, owing to the extraordinary high price of paper, and the unusual quantity of matter to be printed, during the past year, the cost of publication has been greater than usual. 300 copies were published, at a cost of $219.76. Of this sum, $111.95 have been paid by the Treasurer, leaving a balance, unpaid, of $107.81. There are now, in the hands of the Secretary, from 50 to 150 copies of the Transactions, for each year, since 1856. But all printed copies of the Constitution and Bye-Laws, belonging to the Society, are exhausted, and should be republished, together with the Code of Ethics, and a complete list of members. All of which, is respectfully submitted.

N. S. DAVIS,
*Permanent-Secretary.*

Dr. J. H. Hollister, Treasurer, presented the following report, which, with the accompanying vouchers, was referred to an auditing committee, consisting of Drs. Hewins, Rogers, and Jewell:—

J. H. Hollister, *Treasurer*,

In acc't with Ill. State Med. Society,

| | | |
|---|---|---|
| 1864. | *Dr.* | |
| May 3. | —To Cash received from seventeen new members for initiations at date, | $34 00 |
| | Cash for annual dues for 1864, from fourteen members of the above, see memoranda A., | 42 00 |
| | To Cash received from thirty-one permanent members, for annual dues for 1864, see memoranda B., | 93 00 |
| | To Cash received for back dues, for 1863, see memoranda C., | 4 00 |
| | | $173 00 |
| 1864. | *Cr.* | |
| May 3. | —Paid for printing handbills and blanks, (voucher No. 1.) | 5 00 |

| | | |
|---|---|---|
| May 10. | Paid, on order of Committee of Publication, the bill for printing Transactions for 1863, (voucher No. 2.) | 56 05 |
| July 28. 1865. | Paid on order of same, on acc't of printing Transactions for 1864, (voucher No 3.) | 50 00 |
| April 8. | Paid on order of same, on acc't of same, (voucher No. 4.) | 61 95 |
| | | $173 00 |
| | The amount paid to balance former indebtedness, | $56 05 |
| | The amount paid on current expenses of the current year, | 116 95 |
| | Balance now due, for printing Transactions for 1864, | 107 81 |

Respectfully submitted.

J. H. HOLLISTER, *Treasurer.*

On motion, the annual assessment for the year 1865 was fixed at three dollars, for each member.

On motion, the Society adjourned until 7½ o'clock P.M.

EVENING SESSION.

On the assembling of the Society, at 7½ o'clock P.M., the hall was thoroughly filled, with an intelligent audience of citizens to hear a public address from Dr. N. S. Davis, of Chicago. The President called the meeting to order, and introduced the speaker, who delivered an address on The Nature of Medical Science, and its Relations to the Community. The address was listened to with marked attention; and, after its close, the audience retired, and the Society resumed the ordinary transaction of business.

On motion of the Chairman of the Committee of Arrangements, 10 o'clock, on Wednesday morning, was set apart, as the time for visiting the State Normal University.

On motion of Dr. McArthur, the Society agreed to meet at 8 o'clock, the following morning.

Dr. C. Goodbrake moved that the thanks of the Society be tendered to Dr. Davis, for his address, and that a copy be requested for publication.

Dr. F. B. Haller moved, as an amendment, that as soon as published in the Transactions, each member of the Society should endeavor to procure its publication in the leading newspaper of his section of the State.

The amendment was accepted, and the motion, as amended, was adopted.

The Society was invited to hold its next Annual Meeting in Chicago, and also in Decatur. After some discussion, Decatur was selected as the place for holding the next Annual Meeting.

The following gentlemen were regularly proposed, and unanimously elected as permanent members:—

M. B. Skinner, of Mokena, Will Co.
Josiah Brown, of Decatur, Macon Co.
A. McBride, do do.
J. C. Corbus, of Mendota, LaSalle Co.
J. Lehmann, of Bloomington, McLean Co.
Henry Conkling, do do.
H. C. Luce, do do.
J. W. Waters, of Selma, McLean Co.

Dr. Addison Niles, of Quincy, delegate from the Quincy Medical Society; and Dr. S. W. Noble, of Bloomington, permanent member, appeared, and took their seats.

On motion of L. T. Hewins, the Secretary was instructed to procure a suitable book, in which to enter the Constitution, and the names of all the members who sign the same.

Dr. McArthur moved that the Treasurer be instructed to report the list of members who shall be delinquent in the payment of their dues, from year to year.

After some discussion, by Drs. Rogers, Steele, and Davis, the motion was lost.

On motion, the Society adjourned.

WEDNESDAY MORNING SESSION.

The Society was called to order by the President, at 8 o'clock A.M.

On motion of Dr. A. L. McArthur, of Joliet, Drs. H. Noble, J. H. Hollister, and M. B. Skinner, were appointed a committee to prepare resolutions, expressive of the feelings of the Society, concerning the assassination of the late President of the United States.

The report on Ophthalmology, being the special order, Dr. E. L. Holmes, Chairman of the Committee on that subject, presented an interesting report, which may be found in its proper place.

After some comments on that part of the report relating to the application of pressure in the treatment of severe conjunctivitis, and the use of muriate of ammonia in the treatment of iritis, by Dr. Davis; and the relation of a case of paraphlegia, with total blindness, by Dr. Ballard, the report of Dr. Holmes was referred to the Committee of Publication.

The Secretary read a letter from Dr. D. Prince, of Jacksonville, stating that the report on Orthopedic Surgery was completed, but he was detained from attending the meeting of the Society, by the severe illness of his mother.

On motion, the report of Dr. Prince was referred to the Committee of Publication.

The Committee on Nominations then reported the following Committees for the ensuing year:—

STANDING COMMITTEES.

*Committee of Arrangements.*—E. W. Moore, S. McBride, S. T. Trowbridge, J. Brown, and E. S. Jones. W. J. Chenowith, Assistant-Secretary.

*Committee on Practical Medicine.*—H. Noble, J. S. Jewell, F. B. Haller.

*Committee on Surgery.*—A. L. McArthur, L. T. Hewins, S. T. Trowbridge.

*Committee on Drugs and Medicines.*—F. R. Payne, T. D. Fitch, R. E. McVey.

*Committee on Obstetrics.*—C. Goodbrake, W. H. Veatch, C. R. Park.

SPECIAL COMMITTEES.

*On Bromine and its Compounds as Remedial Agents, and the*

*Pathological Conditions in which its Use is indicated.*—Dr. J. H. Hollister, of Chicago.

*On Ophthalmology.*—Dr. E. L. Holmes, of Chicago.

*On Paralysis, its Causes, Pathology, and Principles of Treatment.*—Dr. N. S. Davis.

The hour of 10 o'clock having arrived, a recess was taken to visit the Normal University. Mr. Edwards, the Principal, together with the faculty and teachers of the University gave the Society a very cordial reception; and, after a pleasant interchange of sentiments between Dr. Davis, in behalf of the Society, and Mr. Edwards, the company were conducted through the building, museum, &c., and returned highly gratified with their visit.

AFTERNOON SESSION.

The Society was called to order by the President at 1½ o'clock P.M.

Dr. N. Wright, of Chatham, read a paper on the Importance of Diuretics in the Treatment of Malarious Diseases, which was referred to the Committee of Publication.

Dr. Addison Niles, of Quincy, read the report of a case of Puerperal Fever, successfully treated, chiefly, with large doses of opium. The report was accepted, and referred to the Committee of Publication.

On motion of Dr. S. W. Noble, the Special Committees mentioned on pages 10 and 11 of the printed Transactions for 1864, were continued.

The following members were elected to represent the Society, as delegates, in the next Annual Meeting of the American Medical Association:—

A. L. McArthur, M.D., of Joliet.
L. T. Hewins, M.D., of Loda.
J. W. Redden, M.D., of Shawneetown.
J. M. Steele, M.D., of Grandview.
J. S. Jewell, M.D., of Chicago.
H. Noble, M.D., of Heyworth.
T. F. Worrell, M.D., of Bloomington.
E. L. Holmes, M.D., of Chicago.

J. W. Freer, M.D., of Chicago.
E. W. Moore, M.D., of Decatur.
A. Niles, M.D., of Quincy.
A. W. Heise, M.D., of Joliet.
R. E. McVey, M.D., of Waverly.
L. Clark, M.D., of Rockford.
H. C. Luce, M.D., of Bloomington.
D. Prince, M.D., of Jacksonville.
D. Brainard, M.D., of Chicago.
W. A. Elder, M.D., of Bloomington.
J. Brown, M.D., of Decatur.
N. Wright, M.D., of Chatham.

The following resolutions were offered by the Secretary, and unanimously adopted:—

*Resolved*, That the thanks of the Society be tendered to the Committee of Arrangements for the excellent manner in which they have attended to their duties; and for the hospitalities extended to us during the present meeting.

*Resolved*, That our visit to the State Normal University was highly gratifying to every member, and we regard the institution, under the direction of its present able Principal and Faculty, one of the most important educational institutions in our State.

Dr. H. Noble, in behalf of the Committee appointed for that purpose, made the following report, which was unanimously adopted:—

Mindful of the deep gloom that rests upon all loyal hearts, as the remains of our lamented President are being borne through our midst, to their last resting place; cherishing with inseparable affection with them all that is dear in home and a united land; ready at all times to render personal services and sacrifices for their preservation and defence, second to those of no other class of men, we, the members of the Illinois State Medical Society, in Annual Session convened, desire to make record of our regard for our deceased Chief Magistrate, and the causes that led to his assassination, in the following resolutions:—

1*st*. *Resolved*, That in the death of ABRAHAM LINCOLN, President of the United States, we mourn the loss of an able and pure patriot, a man of the people, allied to them by every tie of interest and affection, earnestly devoting every energy of his being to the integrity and welfare of the entire Union, stricken down by a ruthless and dastard hand, aimed not at himself but the heart of the American Republic.

2*d*. *Resolved*, That in the assassination of the President are manifest the legitimate fruits of that spirit which gave birth to and fostered the rebellion. That to indifferent opposition, and even expressions of encouragement from portions of the Northern press, and the too great leniency of all our people toward treason and traitors, have rendered us all, in a measure, guilty of this mournful result.

The President is slain, but the Government still survives; and we here pledge to it a zealous and resolute support in all measures necessary to arrest and punish treason. We pledge ourselves to sustain our Government with strong arms.

3*d*. *Resolved*. That we commend to the favorable consideration of the American youth, the life and services of our Lamented President, as a brilliant evidence of what are possibilities within the reach of the humblest child of our Commonwealth.

H. NOBLE, *Chairman*,
J. H. HOLLISTER,
M. D. SKINNER.

On motion, the Society then adjourned, to meet in Decatur, on the first Tuesday in May, 1866.

N. S. DAVIS, *Permanent-Secretary*.

